# The Effect of Lumbar Disc Herniation on Spine Loading Characteristics during Trunk Flexion and Two Types of Picking Up Activities

**DOI:** 10.1155/2017/6294503

**Published:** 2017-06-11

**Authors:** Shengzheng Kuai, Weiqiang Liu, Run Ji, Wenyu Zhou

**Affiliations:** ^1^Department of Mechanical Engineering, Tsinghua University, Beijing, China; ^2^Biomechanics and Biotechnology Lab, Research Institute of Tsinghua University in Shenzhen, Shenzhen, China; ^3^National Research Center for Rehabilitation Technical Aids, Beijing, China; ^4^Department of Orthopedics, Shenzhen Second People's Hospital, Shenzhen, China

## Abstract

The main purpose of this study was to investigate the compensatory response of the muscle activities of seventeen major muscle groups in the spinal region, intradiscal forces of the five lumbar motion segment units (MSUs), and facet forces acting on the ten lumbar facet joints in patients with lumbar disc herniation (LDH). Twenty-six healthy adults and seven LDH patients performed trunk flexion, ipsilateral picking up, and contralateral picking up in sequence. Eight optical markers were placed on the landmarks of the pelvis and spinal process. The coordinates of these markers were captured to drive a musculoskeletal model to calculate the muscle activities, intradiscal forces, and facet forces. The muscle activities of the majority of the seventeen major muscle groups were found increases in LDH patients. In addition, the LDH patients displayed larger compressive forces and anteroposterior forces on all the five lumbar MSUs and more lumbar facet inventions on most facet joints. These findings suggest that the LDH patients demonstrate compensatory increases in the most trunk muscle activities and all spinal loads. These negative compensatory responses increase the risk of the aggravation of disc herniation. Therefore, treatment should intervene as earlier as possible for the severe LDH patients.

## 1. Introduction

Low back pain (LBP) is a major health problem that has an enormous effect on many people especially on those who are sitting for prolonged periods. The patients with LBP usually alter their motion patterns to compensate for limited functional motion through different strategies [1]. This alternation may cause local or global musculoskeletal overload which is believed to play a causative role in exacerbating the back disorders or pain [2, 3].

Trunk flexion is a major component of many activities of daily living (ADLs) and is also a routine examination program in the clinical evaluation of LBP [4]. Picking up an object from the floor is also common, but harder, functional activity and may reveal more compensatory strategies in LBP patients. Studies about the two activities have mainly focused on the kinematic analysis including the ranges of motion (ROM) in the lumbar, pelvis, and hip [1, 5–7] and the rhythm between lumbar and hip [8, 9] or between lumbar and pelvis [10–12].

Relevant kinetic studies during the trunk flexion and picking up activities have been limited to the people without back pain [13–15]. Two studies [16, 17] on healthy people have found that the spinal loads and muscle activities would be altered when they were required to change their lumbar rhythm subjectively. Patients with LBP usually adjust their lumbar rhythm due to pain, which may increase the spinal loads and muscle forces and therefore place their trunk system in higher risks of back disorders, tissue injuries, and fatigue.

However, most LBP patients' kinetic studies related to the loads acting on the lumbar region have mainly focused on the different kinds of lifting [18–21] and sit-to-stand [22, 23]. It has been found that LBP patients demonstrate greater compressive forces and shear forces acting on the lumbar region during lifting. Nonetheless, to the authors' knowledge, none of previous reports from the literature have explored the effect of LBP on compressive force and shear force of every lumbar motion segment unit (MSU) during ADLs such as trunk flexion and picking up.

Apart from the intradiscal forces, the muscle forces and activities are also affected by LBP in previous studies. Yahia et al. [24] have found that LBP patients display the deficit of trunk muscles, especially in the extensors when carrying out the isokinetic evaluation. Dubois et al. [25–27] have also reported that LBP would induce increases in lumbar erector spinae (ES) activities during functional tasks.

Facet joint (FJ) is a part of the three-column structure of the vertebrae and plays an important role in load transmission and maintenance of the stability of the spinal motion. In previous kinetic studies during ADLs, the FJ was usually not taken into consideration. However, the facet orientation in the lumbar region has been found irregular alteration in lumbar disc herniation patients [28]. Also, the facet forces have been found amplification under excessive physiological loads that could be induced by LBP [29].

LBP includes a variety of subtypes. The kinematic difference has been found among different subgroups of LBP patients during different ADLs [12, 30, 31]. Likewise, the muscle activation patterns have also been found to be heterogeneity among LBP individuals [32].

Thus, the purpose of this study was to investigate the impact of LBP caused by lumbar disc herniation (LDH) on intradiscal forces and facet forces at five lumbar MSUs and the activities of the eight back main muscle groups and nine front main muscle groups in the spinal region during trunk flexion, ipsilateral picking up, and contralateral picking up. We explored three hypotheses: (1) there were more back muscle activities and less front muscle activities in LDH patients; (2) in the lumbar pathological region, the compressive forces decreased while the anteroposterior shear forces increased in patients with LDH; and (3) there were more interventions of facet joint in the LDH group.

## 2. Method

### 2.1. Subject

Twenty-six healthy adults (mean age 23.6 ± 1.92 years, mean height 169.9 ± 5.9 cm, mean weight 63.5 ± 8.4 kg) and seven LDH patients (mean age 28.7 ± 4.5 years, mean height 170.1 ± 3.4 cm, mean weight 67.4 ± 5.3 kg) participated in this study. The inclusive criteria of the healthy group were (a) no visible motor dysfunction, (b) no any kinds of surgery within recent one year, (c) no any back pain, and (d) no intense exercise 24 hours before trial. The enroll criteria of LDH patients were that (a) the patients were diagnosed with lumbar disc herniation in the course of discopathy in lumbar spine. The diagnosis was made by at least two specialist orthopedic surgeons and confirmed by X-ray imaging and MRI. (b) The disc herniation was diagnosed to occur at the lower lumbar level by MRI. (c) The patients had the ability to conduct level walking and stair climbing. In the examination, the patients were required to attempt to walk and climb stairs. They were deemed to maintain the movement ability if they could perform at least 20 gait cycles. In this study, the disc herniation was found to happen at L4L5 level in three-seventh cases, at L5S1 level in another three-seventh cases, and at both L4L5 and L5S1 levels in one-seventh cases. This study was approved by the Department of Orthopedics of Shenzhen Second People's Hospital in China. All the participants were given informed consent before trial.

### 2.2. Protocol

**Figure 1:**
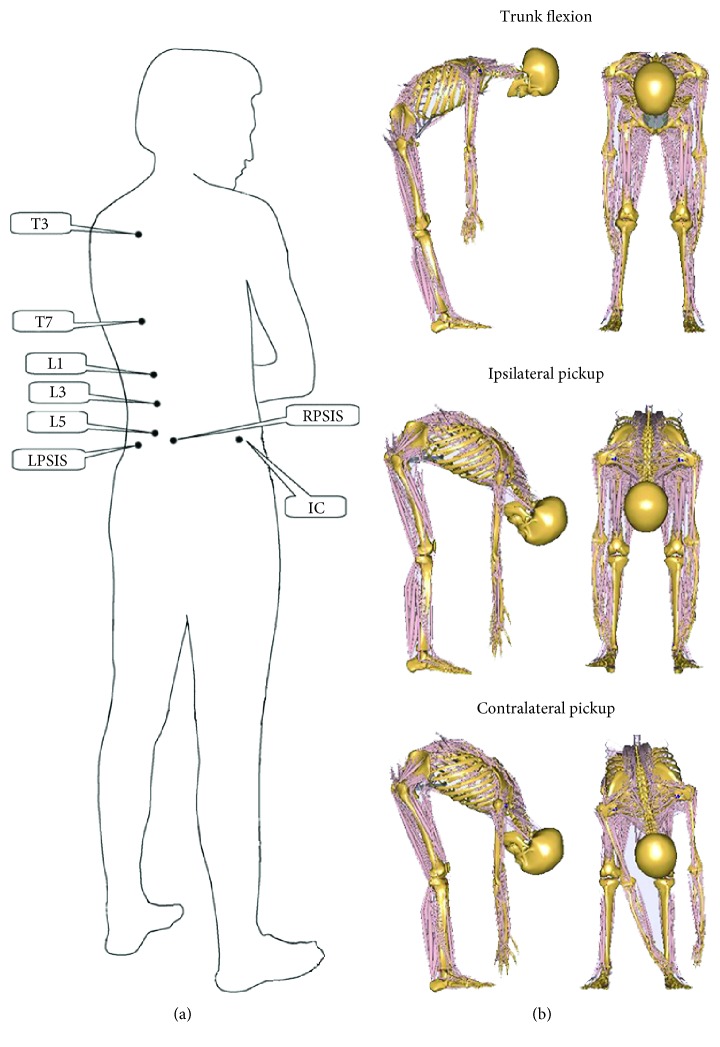
(a) Schematic of the marker placement. (b) Schematic of the test procedure.

The subjects lay in the prone position on a bed, and one surgeon helped to locate the landmarks of the spinous processes of the third and seventh thoracic vertebra (T3, T7), of the first, third, and fifth lumbar vertebra (L1, L3, L5), left and right posterior superior iliac spine (LPSIS, RPSIS), and the iliac crest (IC). Then eight 3D active optical markers were placed on these landmarks (Figure 1(a)). The Optotrak Certus motion analysis system (Northern Digital Inc., Ontario, Canada) was applied to capture the motion of these optical markers at the sample rate of 100 Hz.

Before trials, one surgeon demonstrated the activities of trunk flexion, ipsilateral picking up, and contralateral picking up (Figure 1(b)) and then guided them to practice the three activities several times until they felt they could perform every activity naturally. Every trial was repeated three times for data collection.

### 2.3. Testing Procedure

Before the trial, the participants maintained a neutral upright standing position for at least five seconds to collect the data of baseline. Then, they flexed forward to their maximum voluntary rotation and subsequently returned to their initial position. During the process, the subjects were asked to keep their knee extended. After finishing the task of trunk flexion, the subjects were allowed to take a no more than ten-minute rest. Next, the subjects picked up a small adhesive tape two hundred millimeters in front of their right foot using their right hand. During this ipsilateral picking up activity and subsequent contralateral picking up activity, the subjects were encouraged to give priority to flex their trunk. Likewise, the subjects would have a rest. Afterward, they picked up the object in front of their left foot using their right hand. During the two types of picking up, the subjects were not encouraged to flex their knees except that some subjects could not finish the task without knee flexion.

### 2.4. Musculoskeletal Model and Simulation

A generic FacetJointModel model in the Anybody managed model repository (AMMR, version 1.6) of Anybody modeling system (Anybody modeling system version 6.0.6, Aalborg, Denmark) was applied to calculate the muscle activities, intradiscal forces, and facet forces. This inverse dynamic analysis software and the model were selected since it could quickly predict the forces in a redundant system. In addition, this model has been validated in terms of the minimum-maximum optimization algorithm [33] which was used to solve the recruitment problem. A detailed description of the model has been previously reported and developed by de Zee et al. [33, 34]. In brief, the spinal region consisted of the cervical, thoracic, and lumbar spines. The cervical and thoracic segments were modeled as a single lumped segment. The lumbar spine included five rigid bodies. These segments were connected with an intervertebral joint which was modeled as a spherical joint. The location of each joint was based on the work by Pearcy and Bogduk [35].

The muscles in the model were divided into several functional fascicles. The following muscle fascicles were involved in this spine model: 5 transversus, 3 spinalis, 1 rectus abdominis (RA), 58 erector spinae (ES), 38 lumbar multifidus, 24 thoracic multifidus, 12 oblique externus, 12 oblique internus, 22 psoas major, 10 quadratus lumborum, and 18 semispinalis. All the muscles fascicles were solved as force component in the redundant model system and could only exert tensile force [33, 36, 37].

The model also included ten facet joints in the lumbar region. The location of each facet joint was modeled as a node in the center of the facet contact side on each vertebra, and the orientation of each facet joint was based on the work by Masharawi et al. [38]. The facet force was determined by the distance between the superior and inferior articular facet points of the adjacent vertebra. The contact force solved by this model has been validated based on previous studies [39].

In the Anybody system, the model was driven by the default coefficient of spinerhythm. To investigate the abnormal kinetic characteristics in LDH patients, the model was developed and driven by the captured markers placed on the landmarks of the selected segments. In this study, the motions of L2, L3, and L4 were determined by attributing different weights to the captured markers (Table 1). The other two lumbar segments (L1, L5) were driven using the default ratio of coefficient of spinethythm between L1L2Jnt and L2L3Jnt and between L4L5Jnt and L5S1Jnt, respectively. The pelvic segment was driven by the three markers on the landmarks of the pelvis, and the motion of thoracic segment was determined by the two markers on the thoracic landmarks.

### 2.5. Data Analysis

The excursion angle of the thoracic segment with respect to the baseline was recorded. The analyzed period defined the onset as the moment when the excursion firstly reached the three degrees and the termination as the moment when the excursion firstly raised at the ninety degrees or maximum excursion angle in the condition that the subjects could not flex their trunk over ninety degrees. The maximum muscle activities of the seventeen main muscle groups and the intradiscal forces were analyzed with respect to every excursion angle and normalized to 0–90 degrees with ninety-one points. In addition, the intradiscal forces were also normalized to the weight of every subject.

In the model, the facet force was zero when there was no contact between the superior and inferior articular facet points of the adjacent vertebra. In this study, less than 10 N force was regarded as no contact and more than 50 N force was considered as a strong contact. The facet force between the two threshold values was deemed to be weak contact. Thus, in the present study, the facet intervention includes two levels, namely, the small level that expresses the weak contact and the large level that expresses the strong contact. The durations of small level and large level in the whole period were counted, respectively. Independent group *t*-tests were applied to analyze the difference between healthy subjects and LDH patients on the durations of facet contact in two levels. Data analysis were performed using a custom-made program implemented in MATLAB (The MathWorks, Inc.).

## 3. Results

### 3.1. The Activities of the Main Front and Back Muscle Groups in the Spinal Region

**Figure 2:**
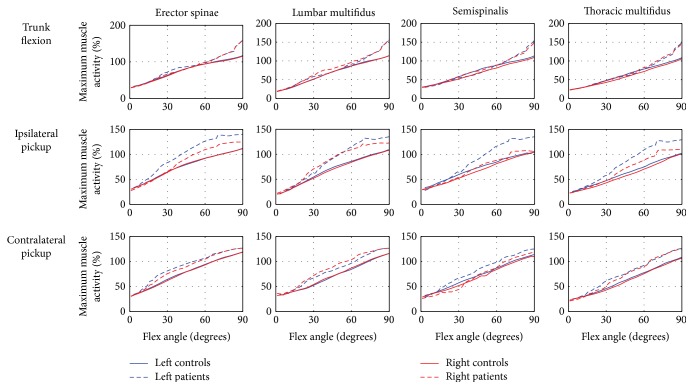
The comparison of the maximum muscle activities of the back main muscle groups in the spinal region between controls and LDH patients. The red and blue solid line—maximum muscle activity in controls; the red and blue dash-dotted line—maximum muscle activity in LDH patients.

**Figure 3:**
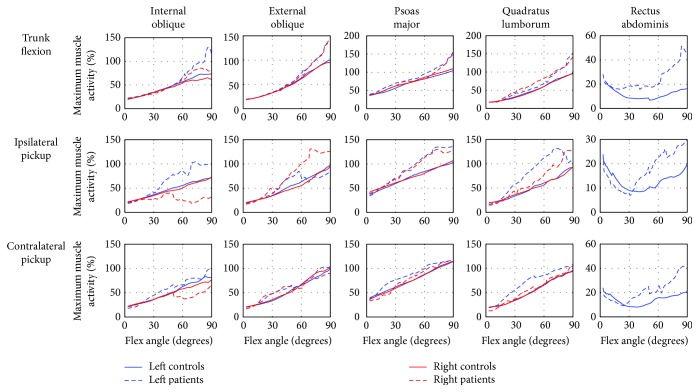
The comparison of the maximum muscle activities of the front main muscle groups in the spinal region between controls and LDH patients. The red and blue solid line—maximum muscle activity in controls; the red and blue dash-dotted line—maximum muscle activity in LDH patients.

During the trunk flexion movement, there was a slight tendency towards larger maximum muscle activities of the four back muscle groups (Figure 2) and IO, EO, PM, and QL (Figure 3) in the end range of flexion. Moreover, the RA was consistently in a larger muscle activity comparing with that in the controls. During the ipsilateral picking up movement, the patients demonstrated more muscle activities of the four back muscle groups (Figure 2) and PM, QL, right EO, and left IO of the front muscle groups (Figure 3) with the increasing flexion angle. Similar to that of trunk flexion, patients demonstrated higher muscle activity of RA in the middle and end ranges of ipsilateral picking up movement. During the contralateral picking up movement, the maximum muscle activities of the four back muscle groups in LDH patients were found larger than those in the controls in the middle and end ranges of this movement.

### 3.2. The Intradiscal Forces in the Spinal Region

**Figure 4:**
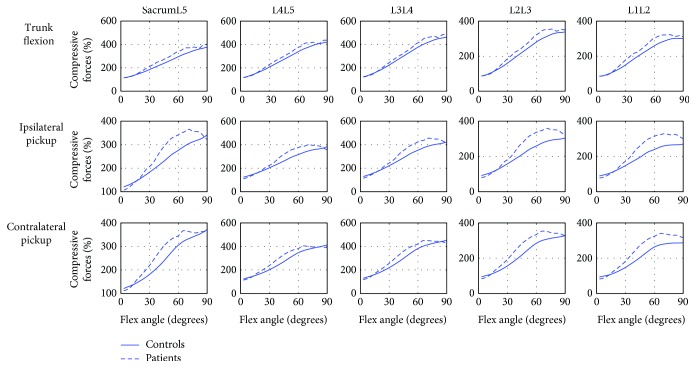
The comparison of the compressive forces in five lumbar discs between controls and LDH patients. The blue solid line—maximum muscle activity in controls; the blue dash-dotted line—maximum muscle activity in LDH patients.

**Figure 5:**
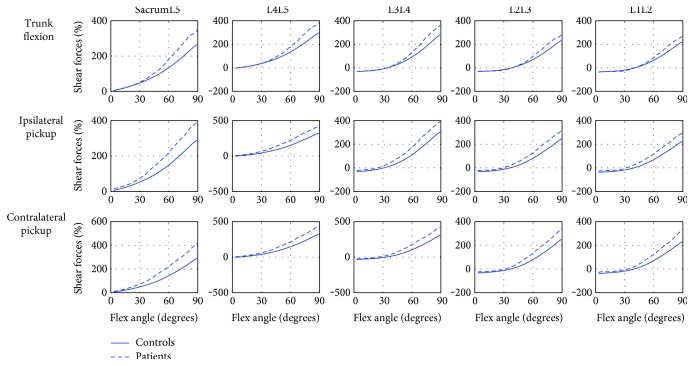
The comparison of the anteroposterior shear forces in five lumbar discs between controls and LDH patients. The blue solid line—maximum muscle activities in controls; the blue dash-dotted line—maximum muscle activities in LDH patients.

The compressive forces and shear forces of all the five lumbar MSUs are shown in Figures 4 and 5, respectively. During the trunk flexion movement, LDH patients demonstrated larger compressive forces on all the five lumbar intervertebral discs (LIDs) with the increase in the flexion angle. During both ipsilateral picking up and contralateral picking up movements, there were significant increases in all the five LIDs in the middle range of the picking up movement in LDH patients. As the flexion angle increasing, the LDH patients demonstrated larger shear forces on all the five LIDs during all the three movements.

### 3.3. The Interventions of Facet Joints in the Spinal Region

**Figure 6:**
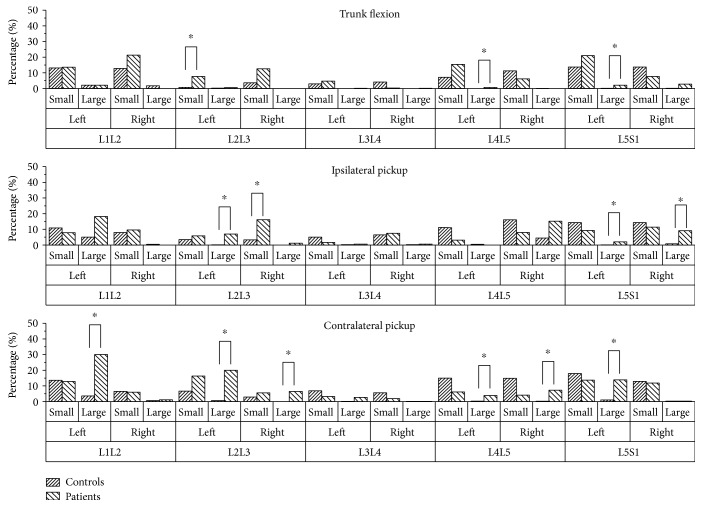
The durations and levels of the facet intervention during the analyzed period. Large represents the sum of duration when the facet forces are more than 50 N; small represents the sum of the duration when the facet forces are between 10 N and 50 N. ∗ indicates the significant difference between controls and patients.

Figure 6 presents the durations of facet intervention on both sides of the five MSUs during the analyzed period. During the trunk flexion movement, LDH patients displayed significantly longer durations of facet intervention on the left facet of L2L3 at a small level, and on the left facets of L4L5 and L5S1 at a large level. During the ipsilateral picking up movement, there were significant increases in the durations of facet intervention on the right facet of L2L3 at a small level and left facets of L2L3, L5S1, the right facet of L5S1 at a large level in LDH patients. In addition, the left facets of L1L2, L2L3, L4L5, and L5S1 and the right facets of L2L3 and L4L5 were found to prolong the durations of facet intervention at a large level significantly during contralateral picking up movement in LDH patients.

## 4. Discussion

This study aims to explore how LDH affect the maximum muscle activities of the seventeen main muscle groups in the spinal region and structural loads acting on every lumbar MSU during trunk flexion and two types of picking up activities.

The maximum muscle activities of all the eight back main muscle groups manifested an increasing tendency at the middle and end ranges of two picking up activities in patients with LDH, supporting the first half of the first hypothesis. However, the maximum muscle activities of all the nine front main muscle groups were not found a reduction in LDH patients which was not consistent with the last part of the first hypothesis. The patients displayed greater compressive forces and anteroposterior shear forces during the three ADLs, which supported the first half of the second hypothesis and disapproved the last half part of the second hypothesis. The more facet inventions were found in patients with LDH during the three ADLs, approving the third hypothesis.

In this study, the muscle activation patterns in LDH patients were not all in consistent with the expectations. In agreement with the previous literature [25–27], LDH patients increased the muscle activities of the back main muscle groups, which may be ascribable to muscle spasm [40] or an attempt to improve spinal stability and protect damaged passive tissue or structure [41–43]. However, the increased muscle activities in the back region did not relieve the muscle activities in the front region in the present study, which was probably because the only increased back muscle activities were not sufficient to compensate for the reduced passive spine stability. Unfortunately, both the increase in the back muscle activities and front muscle activities would lead to a negative consequence because the causative and adaptive relationship between the abnormal muscle activities and lumbar disc herniation development may actually be circular: a higher level of muscle activities predisposes to pain development, following which the muscle activities further increase to alleviate the pain, and the cycle perpetuates.

Compressive forces and shear forces are the two most direct factors that affect the disc herniation [44]. In the third hypothesis, LDH patients should decrease compressive force to reduce the risk of aggravating the disc herniation and increase shear forces as compensation. However, the hypothesis was not validated in this study. The finding showed that both compressive forces and shear forces were greater in LDH patients than in healthy subjects, which was in accordance with prior reports [18, 21, 32]. The larger compressive forces and shear forces were related to excessive muscle coactivity [45], and the increased muscle activities were also found in this study. Moreover, the increase in the intradiscal forces would impose damage to the annulus fibrosus and further induce disc herniation [46].

The facet joint (FJ) is an important structure in lumbar spine and plays a significant role in providing stability to the spinal system. During trunk flexion, small intervention played a predominant role in providing spinal loads. Moreover, five of the ten facet joints were found longer durations of small intervention while only one found shorter duration of small intervention in LDH patients. During ipsilateral picking up and contralateral picking up, the ten FJs mainly demonstrated intervention at a large level. In LDH patients, the durations of large intervention were found significant increases (*P* < 0.05) in three of the ten FJs during ipsilateral picking up and six of the ten FJs during contralateral picking up. Noteworthy was that none of the ten FJs was found a reduction in durations of large intervention. These findings might be due to the restriction in the direction of extension and rotation by FJ [47] and the increased rotation movement from trunk flexion to contralateral picking up activity. The increased FJ intervention in LDH patients might also be a compensatory manifestation of the deficit of soft tissue' stability.

To sum up, it has been suggested that LDH patients displayed more muscle activities, larger intradiscal forces, and more facet interventions during trunk flexion and two types of picking up. These changes might be a compensatory response to relieve pain and improve spinal stability. However, these responses further burdened the trunk musculature, passive soft tissue, and spinal structure during functional tasks. These findings revealed the compensatory mechanism in LDH patients and the necessity of receiving treatments for these patients in terms of spinal loading system.

Some limitations of this study should be noted. First, the sample of patients was relatively small, which might limit the statistical power of the result. Second, only L1, L3, and L5 were placed on optical markers so L1 and L5 could not be driven by captured markers directly. The intersegmental motion difference between L1 and L2 and between L4 and L5 was omitted, which might reduce the difference between two groups in muscle force. Third, the definition of no contact and strong contact of the facet joint was a little arbitrary.

## 5. Conclusions

The present study has shown that LDH patients displayed more muscle activities in the majority of the seventeen main muscle groups in the spinal region, greater compressive forces and anteroposterior shear forces acting on the five lumbar motion segment units, and more facet interventions in the majority of the ten lumbar facet joints. The compensatory response of kinetics in LDH patients played a rather negative role in maintaining the spinal stability and further led to the development of disc herniation. Therefore, patients with severe lumbar disc herniation should receive treatment intervention as early as possible in terms of the compensatory response of muscle activity and spinal load. Moreover, the method in the study will be useful for clinician to assess the biomechanical improvement after different treatments and may also be used towards the development of more effective personalized rehabilitation strategies.

## Figures and Tables

**Table 1:** The weight of markers in trunk section for lumbar vertebrae and thoracic segment.

	Marker T3	Marker T7	Marker L1	Marker L3	Marker L5
Thx	1/2	1/2	0	0	0
L2	0	0	1/2	1/2	0
L3	0	0	1/6	2/3	1/6
L4	0	0	0	1/2	1/2

Thx: the lumped thoracic segment; L1: the first lumbar vertebra; L2: the second lumbar vertebra; L3: the third lumbar vertebra; L4: the fourth lumbar vertebra; L5: the fifth lumbar vertebra; T3: the third thoracic vertebra; T7: the seventh thoracic vertebra.
